# Emergence of Cowpox: Study of the Virulence of Clinical Strains and Evaluation of Antivirals

**DOI:** 10.1371/journal.pone.0055808

**Published:** 2013-02-15

**Authors:** Sophie Duraffour, Barbara Mertens, Hermann Meyer, Joost J. van den Oord, Tania Mitera, Patrick Matthys, Robert Snoeck, Graciela Andrei

**Affiliations:** 1 Rega Institute, Laboratory of Virology and Chemotherapy, KU Leuven, Leuven, Belgium; 2 Bundeswehr Institute of Microbiology, Munich, Germany; 3 Laboratory of Translational Cell and Tissue Research, University Hospitals Leuven, Leuven, Belgium; 4 Rega Institute, Laboratory of Immunobiology, KU Leuven, Leuven, Belgium; Saint Louis University, United States of America

## Abstract

The last years, cowpox infections are being increasingly reported through Eurasia. Cowpox viruses (CPXVs) have been reported to have different genotypes and may be subdivided in at least five genetically distinct monophyletic clusters. However, little is known about their *in vitro* and *in vivo* features. In this report, five genetically diverse CPXVs, including one reference strain (CPXV strain Brighton) and four clinical isolates from human and animal cases, were compared with regard to growth in cells, pathogenicity in mice and inhibition by antivirals. While all CPXVs replicated similarly *in vitro* and showed comparable antiviral susceptibility, marked discrepancies were seen *in vivo*, including differences in virulence with recorded mortality rates of 0%, 20% and 100%. The four CPXV clinical isolates appeared less pathogenic than two reference strains, CPXV Brighton and vaccinia virus Western-Reserve. Disease severity seemed to correlate with high viral DNA loads in several organs, virus titers in lung tissues and levels of IL-6 cytokine in the sera. Our study highlighted that the species CPXV consists of viruses that not only differ considerably in their genotypes but also in their *in vivo* phenotypes, indicating that CPXVs should not be longer classified as a single species. Lung virus titers and IL-6 cytokine level in mice may be used as biomarkers for predicting disease severity. We further demonstrated the potential benefit of cidofovir, CMX001 and ST-246 use as antiviral therapy.

## Introduction

Cowpox virus (CPXV) belongs to the *Orthopoxvirus* (OPV) genus, *Poxviridae* family. Recently, pet rat associated cowpox infections in humans have been reported through Europe with usually mild and self-limiting lesions [1]–[4]. In 1790s, Edward Jenner provided the first exhaustive descriptions of human cowpox in the publication of “An Inquiry Into the Causes and Effects of the VariolaeVaccinae or Cow-Pox (1798)”. Additional reports have further led to the extensive characterization of cowpox illness. Lesions, comparable to those seen with other OPVs, develop from cutaneous papules to vesicules and pustules [1], [3]–[5]. However, severe and/or fatal outcomes have been observed in individuals with impaired immunity such as those suffering of Darier’s disease [6], atopic dermatitis [5], [7], [8], or under steroid therapy [5]. Wild rodents are thought to be the reservoir of CPXV [9], [10].

The recent and numerous cowpox cases in humans have highlighted the difficulties that exist in the differential clinical diagnosis of cowpox and treatment. Since no specific therapy is officially available, antibiotics are given to prevent bacterial infection of lesions. In some cases, due to delayed diagnostic, lesions were surgically excised [1], [3], [4], [11]. However, the off-label use of cidofovir was reported in one clinical case of cowpox [11]. Promising antivirals, although not FDA- or EMA-approved for the therapy of OPV*-*related diseases, are available and might be beneficial for cowpox-related illnesses. They include viral DNA polymerase inhibitors such as cidofovir [Vistide™], requiring intravenous administration, and its lipid derivative CMX001 [HDP-cidofovir], with improved oral bioavailability [12], [13]. ST-246, orally available, inhibits the egress of virus from infected cells [14]. These compounds showed potent antiviral activities against various OPV *in vitro* and *in vivo*, as well as against vaccinia virus (VACV) infections in humans under emergency use [12]–[14].

**Figure 1:**
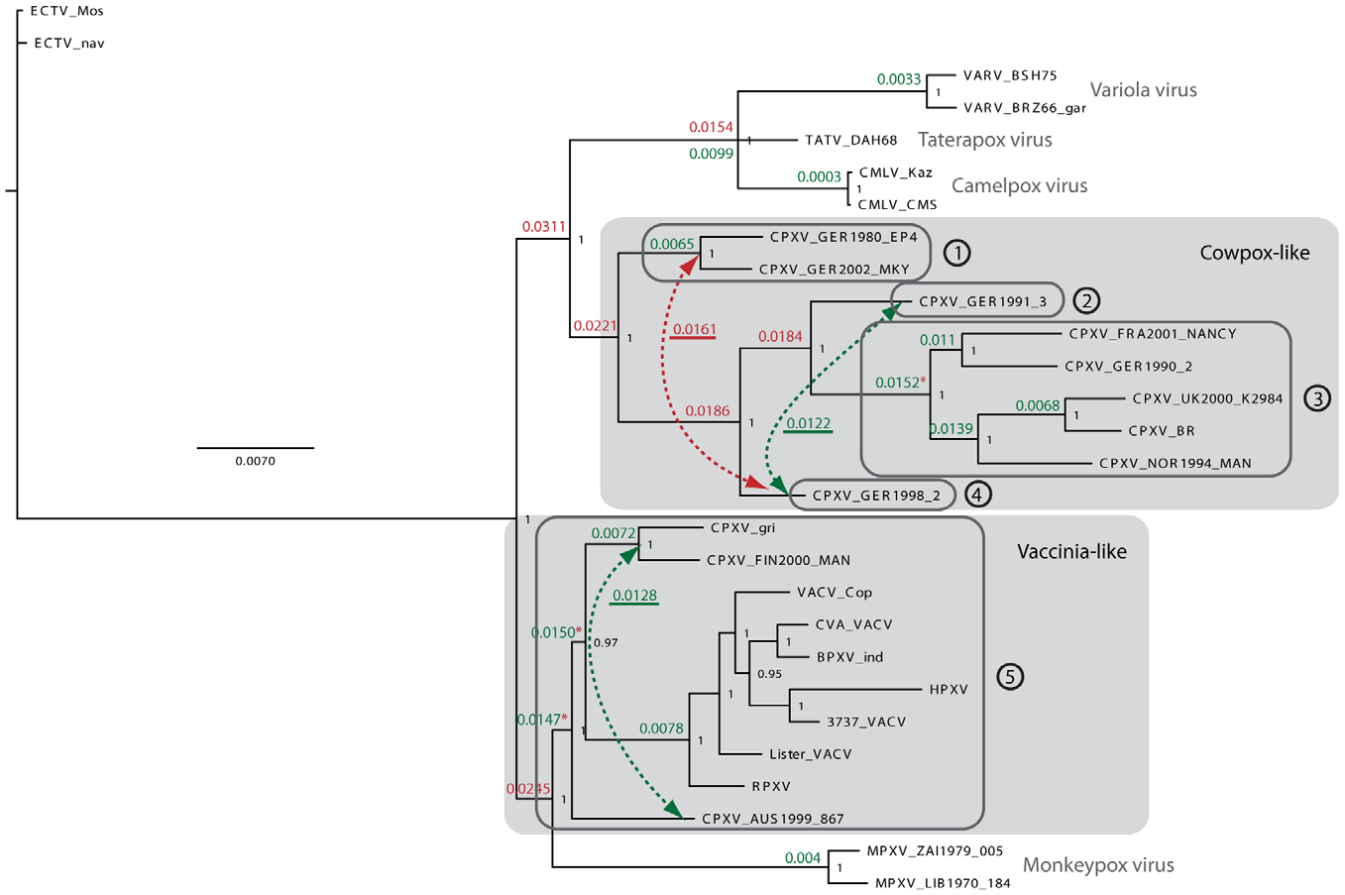
CPXV phylogeny. This figure was reprinted from Carroll et al. [16] under the creative commons license. The tree search was based on alignments of the entire coding regions C23L-B29R of 12 CPXV isolates as described in [16]. The two clades “cowpox-like” and “vaccinia-like” are highlighted, together with the clusters (1 to 5). The virus strains studied here are composed of “cowpox-like” viruses including CPXV-GER-1980-EP4 (cluster 1), CPXV-GER1991-3 (cluster 2), CPXV-BR (cluster 3), and of “vaccinia-like” viruses (cluster 5) including CPXV-FIN2000-MAN and CPXV-AUS1999-867. Although not appearing in the tree, VACV-WR, cluster 5, was also used here.

Our knowledge on the *in vitro* and *in vivo* features of CPXVs originated mainly from work performed with the reference strain Brighton (CPXV-BR). In 1975, Baxby studied the virulence of 18 CPXVs and a remarkable variability in pathogenicity was seen, suggesting a classification in four groups, based on their virulence [15]. Also, recent genotypic data pointed to a much higher genomic diversity among CPXVs as compared to isolates from other OPV species [16]. CPXVs can be grouped into at least two separate, strongly supported and deeply divided clades (**Figure 1**). One clade includes also VACV strains (“vaccinia-like” clade) while the other one includes only strains identified as CPXVs. This “cowpox-like” clade can be further divided into four clusters (**Figure 1**) [16].

Here, we studied the biological properties of five CPXV strains, representing four genetically distinct monophyletic clusters, and their behavior against four antiviral compounds.

## Materials and Methods

### Cells, Viruses and Antivirals

Human embryonic lung fibroblasts (HEL) were used as described in [17]. Five CPXV strains were selected (**Table 1**), three of them, i.e. CPXV-BR, CPXV-GER1980-EP4 and CPXV-GER1991-3, belong to the “cowpox-like” clade, whereas CPXV-AUS1999-867 and CPXV-FIN2000-MAN belong to the “vaccinia-like” clade (**Figure 1**) [16]. For comparison, VACV strains Western-Reserve (VACV-WR), Copenhagen (VACV-Cop) and Lister (VACV-Lis), and camelpox virus (CMLV) strain Iran (CML1) were included [17]. The following compounds were synthesized and kindly provided by Marcela Krečmerová (Academy of Sciences of the Czech Republic v.v.i., Prague, Czech Republic): cidofovir [(*S*)-HPMPC, (*S*)-1-[3-Hydroxy-2-(phosphonomethoxy)propyl]cytosine], (*S*)-HPMP-5-azaC [1-(*S*)-[3-Hydroxy-2-phosphonomethoxy) propyl]-5-azacytosine], CMX001 [HDP-cidofovir, hexadecyloxypropyl-cidofovir]. ST-246 (4-trifluoromethyl-N-(3,3a,4,4a,5,5a,6,6a-octahydro-1,3-dioxo-4,6-ethenocycloprop [f]isoindol-2(1H)-yl)-benzamide) was kindly provided by D.E. Hruby from SIGA Technologies Inc. (Corvallis, OR).

**Table 1:** Cowpox viruses (CPXVs) used in the present study [for references, see (16)].

Strain	Place of isolation	Year	Clinical description	Host	Accession number
CPXV_BR	United Kingdom, Brighton	1937	Local lesions	human	NC_003663
CPXV_GER1991_3	Germany, Munich	1991	Local lesions	human	DQ437593
CPXV_FIN2000_MAN	Finland, Tohmajärvi	2000	Generalized lesions	human	HQ420893
CPXV_AUS1999_867	Austria, Texing	1999	Local lesions	cat	HQ407377
CPXV_GER1980_EP4	Germany, Hameln	1980	Local lesions	elephant	HQ420895

### Antiviral Assays, Cytotoxicity Evaluation and Growth Curves

Experiments were performed in HEL cells as previously described [18], [19].

### Ethics Statement

All animal work was approved by the Katholieke Universiteit Leuven Ethics Committee for Animal Care and Use (Permit number: P044-2010). All animal guidelines and policies were in accordance with the Belgian Royal Decree of 14 November 1993 concerning the protection of laboratory animals and the European Directive 86-609-EEC for the protection of vertebrate animals used for experimental and other scientific purposes. Infections were performed under anesthesia using ketamine/xylazine in saline and, when required, euthanasia was done by administration of pentobarbital sodium.

### Animal Experiments

Female NMRI mice (Elevage-Janvier, Le-Genest-St-Isle, France), 5 weeks old were divided in groups defined as uninfected or as virus-infected with the virus of interest. Mice were inoculated intranasally (i.n.) with 25 µl of phosphate buffer saline (PBS) (uninfected) or with 25 µl of PBS containing 10,000 PFU of the virus of interest (12.5 µl per nostril). Cohorts were monitored for body weight, morbidity and mortality for 30 days. To determine the extent of viral replication, four mice were euthanized at 4 and 7 days post-infection (dpi) and serum as well as various organs were collected as previously described [17]. Organs from one mouse of each group were used for histological examination [17]. Real time quantitative PCR (qPCR) targeting the *F13L* gene was used to quantify viral DNA extracted from sera and tissue samples as previously reported [17]. Sequences of primers and probe for qPCR analysis were as follows: forward primer [5′-CAACTCCATTATAGAAGCAGCCATT-3′], reverse primer [5′-CGTCGTTCTTATCCCAATTACCA-3′] and MGB probe [6-FAM-ATAGAGGAGTTAAGATCAGACTT-MGB]. Virus titers of lung tissue homogenates were determined by titration on HEL cells. ELISA experiments for IL-6 and TNF-α were performed following manufacturer’s instructions (eBioscience, Vienna, Austria) and as previously described [17].

### Statistical Analyses

GraphPad Prism® version 5 Software (La Jolla, CA, USA) was used.

## Results

### Cell Culture Growth and Virulence of CPXVs

In **Table 1** and **Figure 1** are described the virus strains that have been included in this study. Isolates belonging to the “cowpox-like” clade were CPXV-GER1980-EP4, CPXV-GER1991-3 and CPXV-BR, the latter being used as reference strain. The “vaccinia-like” strains included CPXV-FIN2000-MAN, CPXV-AUS199-867 and VACV-WR (reference strain). CPXVs were isolated from different hosts, at diverse time points and in distinct geographic places (**Table 1**).

We first investigated whether CPXV isolates depicted any peculiarities in terms of growth *in vitro*, as compared with CPXV-BR. In cell culture, all CPXVs grew as efficiently as the reference strain CPXV-BR and, albeit CPXV-AUS1999-867 showed a trend of slow growing phenotype, this was not significant (**Figure S1**).

**Figure 2:**
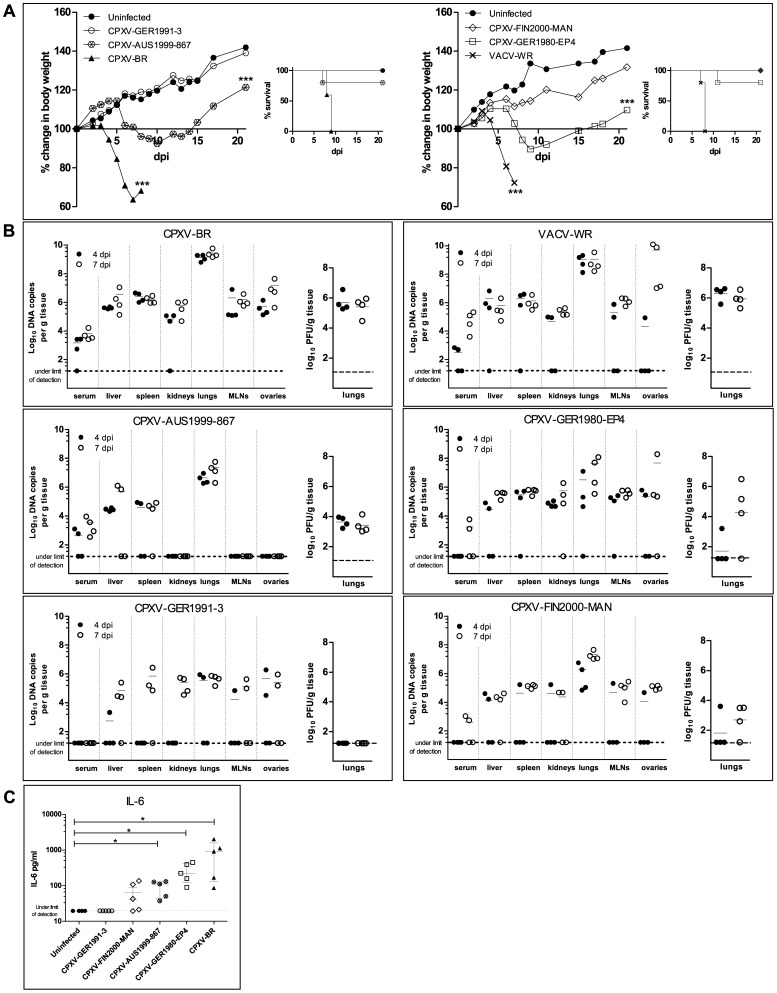
Virulence of CPXVs, virus distribution in tissues and cytokine levels in the sera. Animals were challenged intranasally with 10,000 PFU/mouse with VACV-WR, CPXV-BR, CPXV-GER1980-EP4, CPXV-GER1991-3, CPXV-AUS1999-867 and CPXV-FIN2000-MAN (13 mice per group). (**A**) Body weight evolution, survival curves of each group are given, and are representative of two independent experiments. *** (p<0.001), the body weight of uninfected animals differs significantly from that of infected mice (one-way analysis of variance (ANOVA) associated with a Dunnett’s multiple comparison test). (**B**) Viral loads in sera and organs (left graph) and lung virus titers (right graph) are shown. The virus strain is indicated on top of each graph. Viral loads were determined by qPCR and are expressed as log_10_ DNA copy numbers per 50 µl of serum or per g of tissue for liver, spleen, kidneys, lungs, MLNs and ovaries. Lung virus titers are shown in log_10_ PFU per g of lung tissue. Four individual mice per group and per time point were used. Symbols: 4 dpi (•) and 7 dpi (○), and dashed line represents the limit of detection. (**C**) IL-6 production in the sera of mice is shown. Sera were collected at day 7 pi after exposure to PBS or to virus. Data are the median ± interquartile range (*n*  = 4 or 5 mice for each group). *p = 0.0179, IL-6 level of virus-infected mice differs significantly from that of the uninfected group by Mann-Whitney test.

A mouse model was then used to assess virulence of each CPXVs and of VACV-WR. As depicted in **Figure 2A**, both CPXV-BR and VACV-WR induced marked body weight loss (p<0.001) that ultimately led to 100% mortality by 7 to 9 dpi.

Animals exposed to CPXV-AUS1999-867 and CPXV-GER1980-EP4 showed significant loss of body weight (p<0.01) and 20% of the cohorts died, respectively, at 7 and 11 dpi. Surviving animals recovered progressively from loss of weight after 10 dpi. However, tail lesions were seen between 6 and 12 dpi with CPXV-AUS1999-867, whereas lesions on foot and tail, as well as marked signs of conjunctivitis appeared from 17 dpi with CPXV-GER1980-EP4.

In contrast, mice inoculated with CPXV-GER1991-3 and CPXV-FIN2000-MAN did not show any signs of sickness and there was no significant weight loss. Some tail lesions (pustules) appeared on 13 dpi with CPXV-GER1991-3, but resolved soon.

### Differences in Virus Distribution in Tissues Among CPXV-infected Mice

Four animals of each group were sacrificed at 4 and 7 dpi and viral DNA load was determined in various organs.

**Figure 3:**
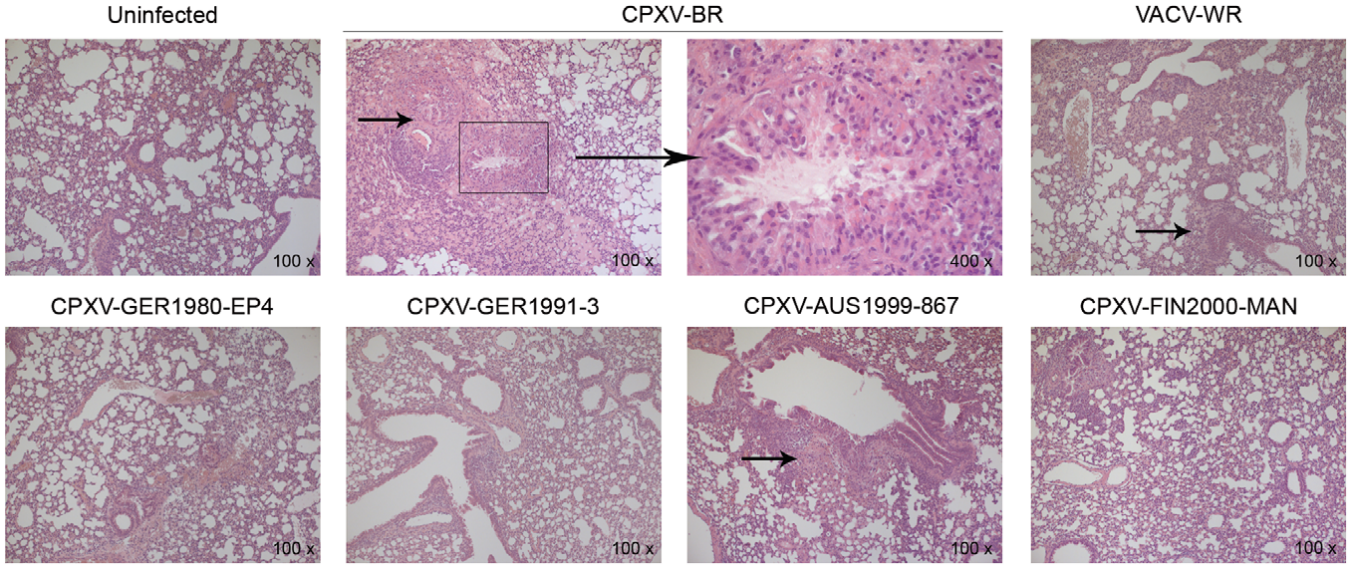
Lung tissue examinations at 7 dpi. While CPXV-BR, CPXV-AUS1999-867 and VACV-WR exhibited pneumonia, interstitial inflammation was observed with CPXV-GER1980-EP4. Many inflammatory cells, sometimes localized around the bronchioli, were noted upon infection with CPXV-FIN2000-MAN. Only few inflammatory cells were seen with CPXV-GER1991-3. The arrows head points at edema and collapse of the lungs. Magnification, 100 ×. Of note, eosinofilic cytoplasmic inclusions in the lung tissue of CPXV-BR-infected mouse are also shown (400 ×).

CPXV-BR DNA was found in all organs as soon as on 4 dpi (**Figure 2B**), with 5 to 9 log DNA copies/g tissue, sera being also positive at these time points, with mean DNA copy numbers of 3.2 and 3.8 log/50 µl serum at 4 and 7 dpi, respectively. The lung tissues gave the highest level of DNA copies, at both days evaluated, with a mean value of 9.3 log DNA copies/g tissue, which was equivalent to a virus titer of 5.5 log PFU/g tissue. Histological examination of lung tissue revealed acute inflammation with alveolar epithelial cells containing eosinophilic cytoplasmic inclusions bodies that are characteristic for CPXV-BR infection (**Figure 3**).

A similar trend of virus spreading was observed with VACV-WR albeit not all organs were positive for DNA at 4 dpi and, at 7 dpi, the viral DNA load in the sera [5 log/50 µl serum] and ovarian tissue [9.7 log/g tissue] were higher than those of CPXV-BR. Virus titers in the lungs reached an average of 6 log PFU/g tissue at both time points and pneumonia was noted (**Figure 3**).

While showing a comparable virulence (**Figure 2A**), infection with CPXV-AUS1999-867 and CPXV-GER1980-EP4 resulted in different viral DNA load profiles. Strikingly, kidneys, mesenteric lymph nodes (MLNs) and ovaries of mice inoculated with CPXV-AUS1999-867 were negative for circulating viral DNA and only the lungs showed an average of 7 log DNA copies/g which corresponded to a mean titer of 3.5 log PFU/g tissue at 4 and 7 dpi (**Figure 2B**). In these animals, the lungs showed pneumonia (**Figure 3**).

Circulating CPXV-GER1980-EP4 DNA was evidenced in most of the tissues analyzed at 4 and 7 dpi, with the exception of the serum at 4 dpi, with mean viral DNA loads of 5 log copies/g tissue. Replicating virus in the lung tissue was only detectable in one [3.2 log PFU/g] out of four animals at 4 dpi, and in three out of four mice at 7 dpi [mean titer of 4.3 log PFU/g]. Interstitial inflammation was noted in lung tissues (**Figure 3**).

The pattern of viral DNA loads observed with CPXV-GER1991-3 was clearly different from that of CPXV-BR and this might be due to impaired growth of CPXV-GER1991-3 in the organs examined. Indeed, at 4 dpi, most of the organs were negative for viral DNA, including liver, spleen, kidneys and MLNs, and only two out of four mice had detectable viral DNA in the lungs and ovaries. At 7 dpi, viral DNA was found in most of the organs, but only kidney [5.4 log/g] and lung [5.7 log/g] tissues were positive in all animals. In lungs, the first organ targeted following i.n. infection, viral DNA was present but no viable virus was detected, either at 4 or 7 dpi (**Figure 2B**). Few foci of inflammatory cells were seen upon histological analysis of the lungs (**Figure 3**).

Most of the tissues were negative for DNA of CPXV-FIN2000-MAN at 4 dpi, with the exception of lung tissue [mean of 6.3 log DNA copies/g] which is in contrast with the observation made with CPXV-GER1991-3 at this time point. At 7 dpi, all the mice showed presence of viral DNA in the spleen, lungs, MLNs and ovaries. Replicating virus in lungs was found in only one [3.6 log PFU/g] out of four animals at 4 dpi and in three out of four mice at 7 dpi [2.7 log PFU/g]. Lung tissue exhibited an increased interstitial cellularity with inflammatory cells that were sometimes localized in the peribronchial area (**Figure 3**).

### TNF-α and IL-6 Production in the Sera

Tumor necrosis factor (TNF-α) and interleukin-6 (IL-6) are pro-inflammatory cytokines, and while TNF-α activates innate responses to infection and promotes an anti-viral state in cells, IL-6 has been described as one of the mediator coordinating the interface between adaptive and innate immunity [20], [21]. CPXVs encode several genes that can counteract the host immune responses, including the inhibition of TNF-induced responses (for review see [20]). Also, IL-6 has been shown to be increased in response to CPXV infection in mice [22], and recently *in vitro* [23] and in macaques [24], [25]. We thus investigated the level of expression of these two cytokines, by ELISA, in sera samples collected on 7 dpi from CPXV-infected animals. In all cohorts examined, TNF-α levels were under the limit of detection (<39.1 pg/ml). Serum IL-6 levels correlated with morbidity (**Figure 2C**). IL-6 concentrations after CPXV-GER1991-3 or CPXV-FIN2000-MAN exposure were similar to those of uninfected animals (p>0.05) whereas increased levels were seen (*p* = 0.0179) following CPXV-BR, CPXV-AUS1999-867 or CPXV-GER1980-EP4 infection (**Figure 2C**).

### Inhibitory Effects of Viral DNA Polymerase and Viral Egress Inhibitors *in vitro*

As the use of antiviral therapy may be beneficial for managing CPXV-related infections, we further investigated the inhibitory activities of four anti-OPV agents against CPXV replication *in vitro* (**Table 2**). Other OPVs, including three strains of VACV and one strain of CMLV were also examined (**Table 3**). Both cidofovir and (*S*)-HPMP-5-azaC [5-aza derivative of cidofovir] inhibited the replication of all OPVs tested in the micromolar range. The molecule CMX001 showed approximately 1000-fold lower EC_50_ values than its parent drug (i.e. cidofovir) and appeared more selective. However, the toxicity of this molecule was higher on growing cells than those of cidofovir and (*S*)-HPMP-5-azaC. The egress inhibitor ST-246 demonstrated comparable inhibitory effects in the nanomolar range against the different viruses and, due to its low toxicity for growing cells, the molecule was highly selective. It has to be noticed that ST-246 EC_50_ value against CPXV-BR was 5- to 10-fold lower than those reported with CPXV clinical isolates, VACV strains and CML1. Nevertheless, ST-246 remained still potently active in the nanomolar range against CPXV-BR.

**Table 2:** Antiviral activities and selectivity indices of anti-*orthopoxvirus* compounds against cowpox (CPXV) strains.

	CPXV-BR^a^		CPXV-GER1980-EP4		CPXV-GER1991-3		CPXV-AUS1999-867		CPXV-FIN2000-MAN	
Compounds^b^	EC_50_ (µM)^c^	SI^d^	EC_50_ (µM)	SI	EC_50_ (µM)	SI	EC_50_ (µM)	SI	EC_50_ (µM)	SI
Cidofovir	19.6±9.8	16	20.6±0.5	15	7.7±2.8	40	13.1±4.8	24	12.2±6.8	25
(*S*)-HPMP-5-azaC	19.3±11.1	6	19.4±2.7	6	7.1±3.0	15	14.1±3.8	8	12.0±5.1	9
CMX001	0.030±0.024	≥71	0.017±0.000	≥123	0.007±0.007	≥300	0.01±0.007	≥191	0.014±0.010	≥150
ST-246	0.21±0.15	≥1,366	0.03±0.04	≥9,567	0.03±0.02	≥9,567	0.02±0.01	≥14,350	0.02±0.01	≥14,350

a Viral strains tested.

b For full chemical names, see Materials and Methods.

c EC_50_ values or 50% effective concentrations are given as the mean ±SD of at least three independent experiments, and were defined as the concentration of compound required to reduce viral cytopathic effect by 50%. Briefly, confluent HEL cells were infected at a multiplicity of infection of 0.01 PFU/cell for 2 h after which residual virus was removed and replaced with fresh medium containing serial dilutions of the test compounds (in duplicate). After 2 to 4 days, viral cytopathic effect was recorded and EC_50_ values were determined.

d SI or selectivity index is the ratio CC_50_/EC_50_. CC_50_ or 50% cytostatic concentration was defined as the concentration required to reduce cell growth by 50%. CC_50_ values were of 301±171 µM for cidofovir, 108±72 µM for (*S*)-HPMP-5-azaC, ≥2.1±1.6 µM for CMX001 and ≥287±176 µM for ST-246.

**Table 3:** Antiviral activities and selectivity indices of anti-*orthopoxvirus* compounds against vaccinia (VACV) and camelpox virus (CML1) strains.

	VACV-WR^a^		VACV-Cop		VACV-Lis		CML1	
Compounds^b^	EC_50_ (µM)^c^	SI^d^	EC_50_ (µM)	SI	EC_50_ (µM)	SI	EC_50_ (µM)	SI
Cidofovir	8.1±4.4	38	5.6±2.8	55	5.9±3.8	53	10.8±5.9	29
(*S*)-HPMP-5-azaC	7.3±1.6	15	3.9±1.8	28	9.9±7.8	11	13.9±9.2	8
CMX001	0.007±0.009	≥300	0.004±0.002	≥525	0.094±0.061	≥22	0.021±0.015	≥100
ST-246	0.017±0.009	≥16,882	0.008±0.003	≥35,875	0.04±0.06	≥7,175	0.02±0.02	≥14,350

a, b, c, d are defined in the footnote of **Table 2**.

## Discussion

Here, we compared the biological properties of five CPXV strains that are representative of four different genetic clusters (**Figure 1**) [16]. Based on viral kinetic analysis, a comparable growth in cells for all CPXVs was demonstrated. However, virulence in mice underlined marked differences between the CPXVs. Depending on the disease outcome, CPXVs could be allocated in three groups characterized by no death, 20% or a 100% mortality rate. Cohorts infected i.n. with CPXV-BR and VACV-WR depicted 100% lethality, with high levels of replicating virus in the lungs and spreading to various organs, which is consistent with published observations [22], [26]. CPXV-BR was originally isolated in 1937 from a milker and maintained by serial passage in rabbit skin [27], and VACV-WR is historically derived from NYCBH by several passages in mouse brain [28]. While we can hypothesize that the virulence of VACV-WR might be explained by its adaptation in mouse brain, the pathogenicity of CPXV-BR in mice might rely on its genetic background (i.e. virulence factors, or immunomodulatory proteins).

Strikingly, two CPXV isolates derived from an elephant (CPXV-GER1980-EP4) and a cat (CPXV-AUS1999-867) induced a 20% mortality rate in contrast to two strains isolated from humans that did not cause mortality. A slight (CPXV-FIN2000-MAN) or no weight loss (CPXV-GER1991-3) was observed. In line with this, Huemer and colleagues demonstrated that a human-derived CPXV was impaired in pathogenicity following intravenous or i.n. infection of BALB/c mice, at virus doses ranging from 10^3^ to 10^7 ^PFU per animal [29].

The variability in virulence among the CPXVs reported here correlates with the observations of Baxby [15]. Eighteen CPXVs, including CPXV-BR, were evaluated by infecting mice intracerebrally (10^5^ PFU per animal) and were classified in four groups based on mortality rates (0%, 15–25%, 55–65% and 90–100% mortality, CPXV-BR being part of the latter group). The authors noted that the variability was unlikely to be explained by a different passage history of the viruses [15], although this was not assessed here. Additionally, it has to be mentioned that our *in vivo* observations relied on mice inoculated with a virus dose of 10^4^ PFU per animal, and this dose was chosen because it is 100% lethal for mice i.n. exposed to either CPXV-BR or VACV-WR reference strains (**Figure 2**). It cannot be excluded that CPXV isolates might be as pathogenic as the reference strains if animals were inoculated with a 10- or 100-fold increased virus dose, but only further investigations will give an answer to that question.

Our results suggested that the virus ability to replicate in the lungs may account for disease outcome, as observed with CPXV-BR, CPXV-GER1980-EP4 and CPXV-AUS1999-867, that had virus titers ≥4 logs PFU/g tissue. Also, elevated concentrations of the pro-inflammatory cytokine IL-6 seemed to correlate with CPXV disease outcome. This result is in line with increased secretion of IL-6 reported in the sera of cowpox-challenged macaques [24], [25]. The role of IL-6 induction in cowpox pathogenesis remains still unclear but a recent article evidenced that IL-6, IL-8 and CXCL1, produced upon *in vitro* CPXV-BR infection, could be responsible for chemotaxis of monocytes *in vitro* [23]. Additionally, macrophages and monocytes are suspected to be involved in CPXV spread through the host [30]. A potential role of IL-6 in CPXV pathogenesis might also be supported by the fact that, during inflammation, IL-6 has been shown to suppress neutrophils recruitment at sites of acute inflammation, making ways for the influx of monocytes as the inflammatory response is sustained [21]. No evidence of systemic TNF-α induction was seen which might not be surprising as OPVs, and CPXV in particular, utilize many ways to counteract TNF-α production through the expression of NFκB inhibitors or of cytokine response modifiers (CrmB, C, D and E) (for review see [20]). Undetectable levels of TNF-α were also reported *in vitro*, following monkeypox virus infection [31], as well as in sera of macaques lethally infected with variola virus [32]. In our hands, both lung virus titers and IL-6 sera levels emphasized differences between CPXV strains. Nevertheless, the absence of spreading of CPXV-AUS1999-867 to kidneys, MLNs and ovaries, and the avirulence of CPXV-GER1991-3 in NMRI mice still require additional studies that might point towards nonfunctional gene(s) needed for efficient virus spreading (CPXV-AUS1999-867), or for immunomodulation or host range (CPXV-GER1991-3).

The discrepancies observed here raised the question whether CPXV-BR should be considered a representative CPXV, as this strain has an unknown passage history in various laboratories since its isolation 80 years ago. It could be that a reduced virulence or an avirulent phenotype in NMRI mice may be a typical feature of CPXV clinical isolates, explaining their ability to survive in rodent populations. The virus has to rely on its host and should therefore not be lethal, but CPXV infections may still have a direct impact on the dynamics of their reservoir hosts. In line with that, infection of bank voles and wood mice with a CPXV strain isolated from a cat have been shown to impair fecundity although any overt disease and mortality were recorded [33], [34]. Also, CPXV infection of field voles reduced significantly their survival rates [35].

Antivirals in clinic could be valuable to manage cowpox infections in both humans and animals. We therefore examined the antiviral activities of promising anti-OPV compounds against CPXVs. Our *in vitro* evaluation evidenced that compounds targeting viral DNA replication, i.e. cidofovir, (*S*)-HPMP-5-azaC and CMX001, inhibited similarly CPXV clinical isolates and the CPXV, VACV and CMLV reference strains. Comparable findings were obtained with the viral egress ST-246, although the EC_50_ value against CPXV-BR appeared 5- to 10-fold higher than that of CPXV isolates, VACV and CML1. These results are in agreement with our previous studies demonstrating that the way of propagation of CPXV-BR in HEL cells had an impact on ST-246 antiviral activity [36]. ST-246 inhibits the wrapping of virions and, as a consequence, inhibits the production of enveloped viruses. However, we have shown that CPXV-BR intracellular mature viruses can still be released in the cell supernatant following ST-246 treatment, and this viral form could most probably account for CPXV-BR EC_50_ value [36]. Our *in vitro* findings together with numerous *in vivo* antiviral studies performed with laboratory strains [12]–[14] strongly support that these compounds will likely be active *in vivo* against genetically diverse CPXV isolates.

In this study, we demonstrate the importance of both genomic and biological testing in characterizing emerging isolates. The extrapolation of these results for the clinicians and veterinarians should be made in function of the immune status of the patient and the virulence of the strain which may give variable clinical pictures. Antiviral treatments (i.e. cidofovir, (*S*)-HPMP-5-azaC, CMX001 and ST-246), specific for managing OPV-related infections, exist and, depending on the clinical case, they could be administered locally or systemically.

## Supporting Information

Figure S1 **All CPXV clinical isolates grew as efficiently as the reference CPXV-BR strain in HEL cells.** Growth curves (left column) and linear regression analysis (right column) are depicted. CPXV-BR growth was compared with CPXV-GER1980-EP4 (A), CPXV-GER1991-3 (B), CPXV-AUS1999-867 (C) and CPXV-FIN2000-MAN (D). HEL cells were infected with the indicated strains at a MOI of 0.01 PFU/cell. The virus was collected at the indicated time points. The results originated from two independent experiments and are presented as means ± the SD. The best-fit lines of the linear regression analysis from 9 to 48 hpi (representative for the linear part of the curve) are shown. The growth rates between CPXV-BR and CPXV clinical strains were subjected to statistical analysis as previously described [19]. Briefly, the mean slope (rate of growth in PFU/ml) and intercept of the virus regression line of CPXV-BR was compared with those of the CPXV clinical strains. The p-values of the slope were all greater than 0.05. Therefore, only the p-values of the intercept are shown. Statistical significance was only considered for p≤0.01.
(TIF)
