# Supplementary figures and images for: Emergence of Cowpox: Study of the Virulence of Clinical Strains and Evaluation of Antivirals
Source: PLoS One. 2013 Feb 15;8(2):e55808. doi: 10.1371/journal.pone.0055808 (PMC3574090; doi:10.1371/journal.pone.0055808)

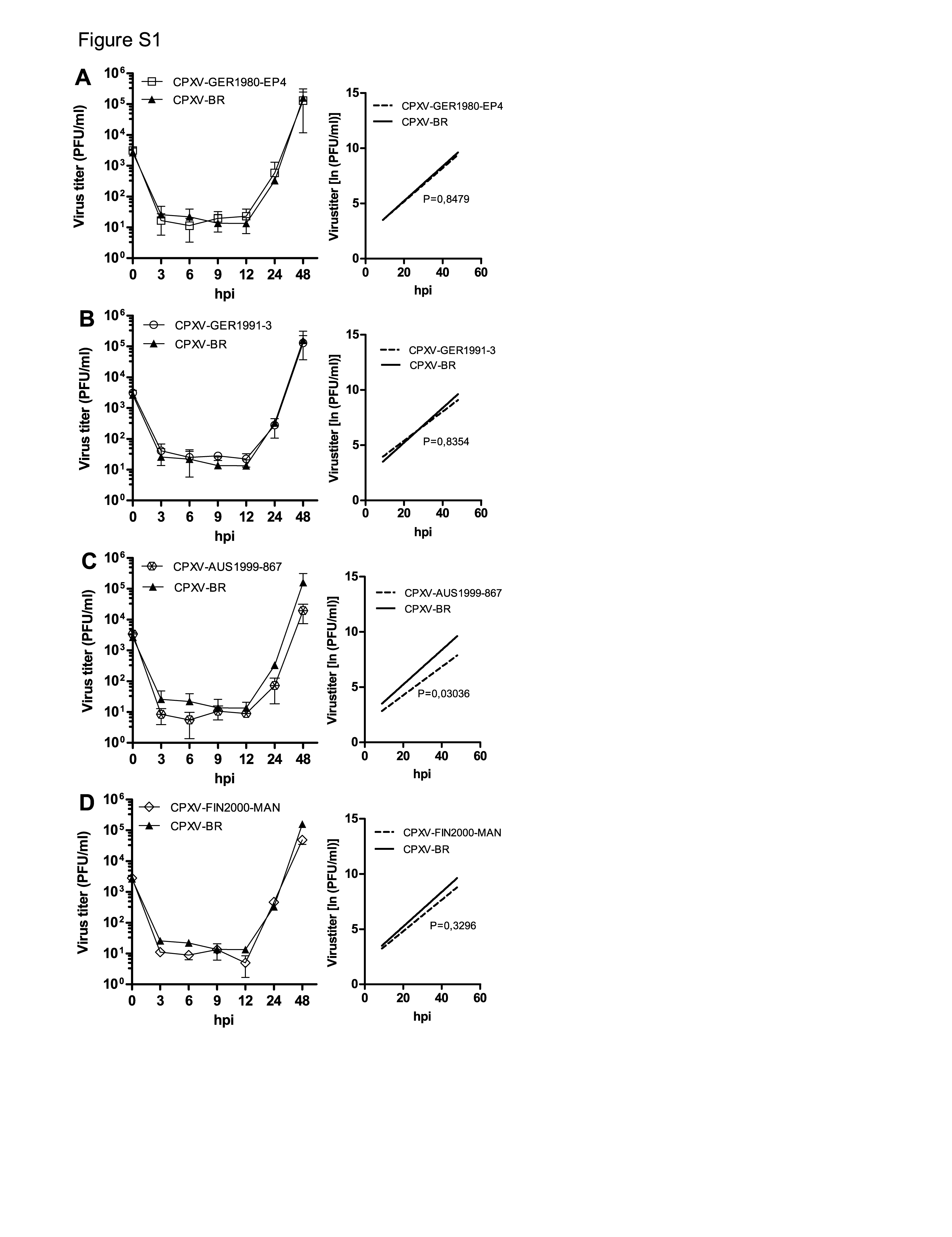

Supplement: Figure S1 — All CPXV clinical isolates grew as efficiently as the reference CPXV-BR strain in HEL cells. Growth curves (left column) and linear regression analysis (right column) are depicted. CPXV-BR growth was compared with CPXV-GER1980-EP4 (A), CPXV-GER1991-3 (B), CPXV-AUS1999-867 (C) and CPXV-FIN2000-MAN (D). HEL cells were infected with the indicated strains at a MOI of 0.01 PFU/cell. The virus was collected at the indicated time points. The results originated from two independent experiments and are presented as means ± the SD. The best-fit lines of the linear regression analysis from 9 to 48 hpi (representative for the linear part of the curve) are shown. The growth rates between CPXV-BR and CPXV clinical strains were subjected to statistical analysis as previously described [19]. Briefly, the mean slope (rate of growth in PFU/ml) and intercept of the virus regression line of CPXV-BR was compared with those of the CPXV clinical strains. The p-values of the slope were all greater than 0.05. Therefore, only the p-values of the intercept are shown. Statistical significance was only considered for p≤0.01. (TIF) [file pone.0055808.s001.tif]
